# Supplementary material for: Chromosome fusions shaped karyotype evolution and evolutionary relationships in the model family Brassicaceae
Source: Nat Commun. 2025 May 19;16:4631. doi: 10.1038/s41467-025-59640-2 (PMC12089291; doi:10.1038/s41467-025-59640-2)
Supplement: Supplementary file 1 — Supplementary Information [file 41467_2025_59640_MOESM1_ESM.pdf]

Chromosome fusions shaped karyotype evolution and evolutionary relationships in the model family Brassicaceae

Jiang *et al.*

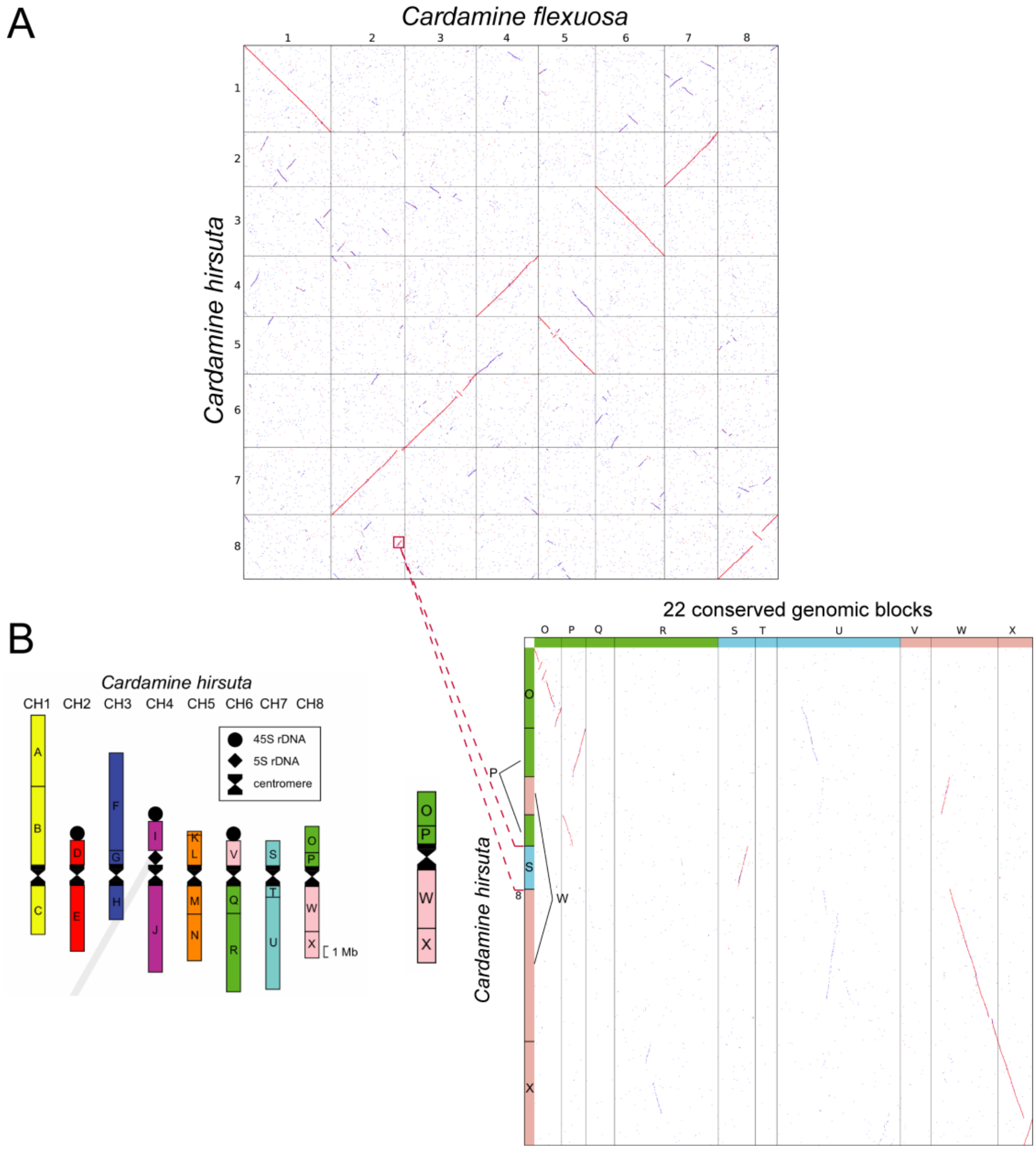

Supplementary Figure 1. *Cardamine flexuosa*'s genome may be assembled from *C. hirsuta*, but with a better accuracy than another genome assembly of *C. hirsuta*. (*a*) The Chromosome Counts Database (https://taux.evolseq.net/CCDB_web/search/) indicates that *C. hirsuta* is diploid (*n* = 8) and *C. flexuosa* is tetraploid (*n* = 16). However, the *C. flexuosa* assembly appears to be a diploid genome, possibly assembled from *C. hirsuta* (Supplemental Data 1). The dotplot between "*C. flexuosa*" and *C. hirsuta* reveals a minor difference only in a small Chr8 segment (marked in red), with all other chromosomes fully collinear. Therefore, we used the "*C. flexuosa*" genome assembly for subsequent analyses. **(*b*)** Comparative cytogenomic map (Figure 2 in Mandáková *et al*.[1]) suggests that the small segment should be on Chr7 rather than on Chr 8 in *C. hirsuta*.

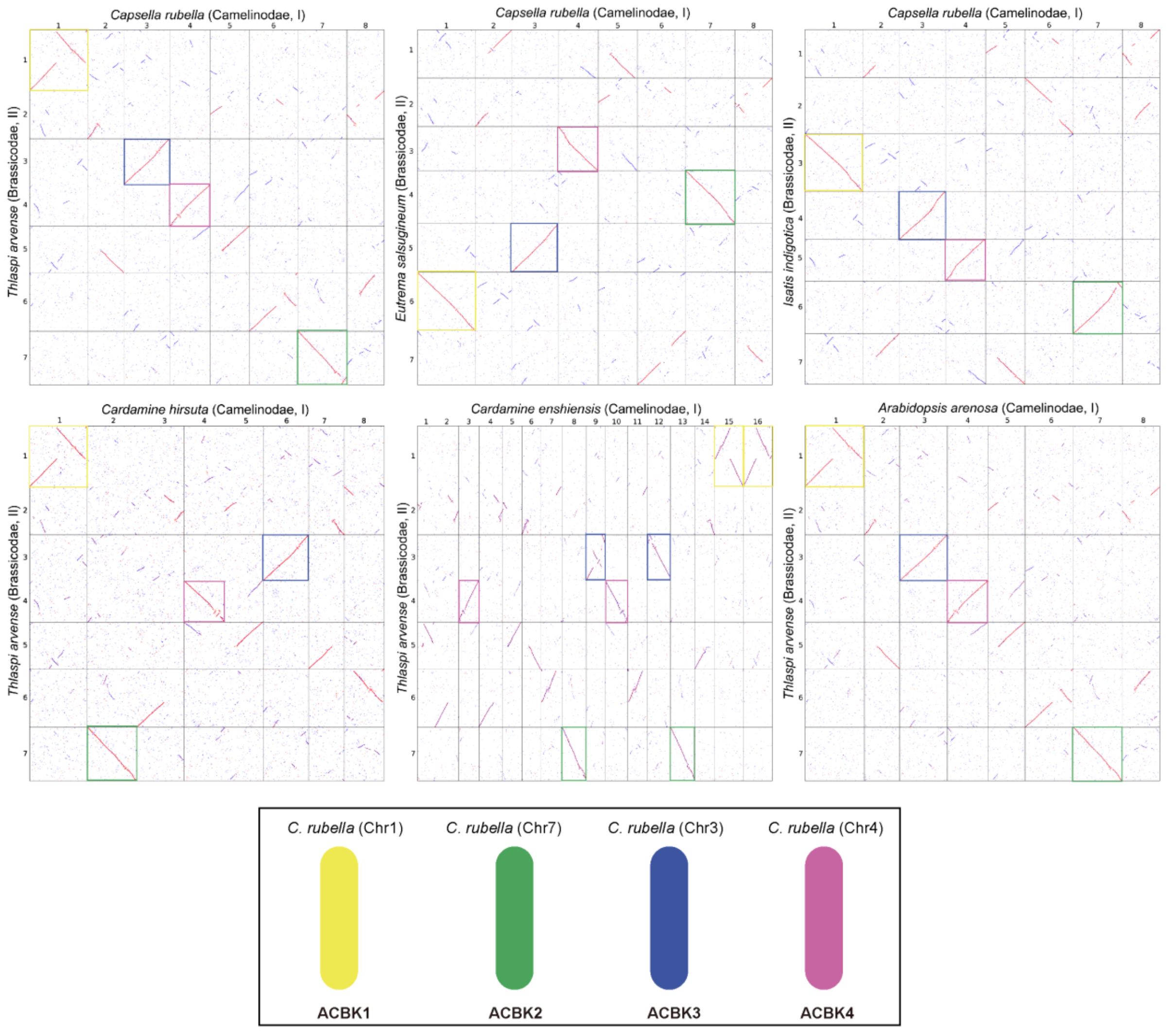

Supplementary Figure 2. Identification of four protochromosomes within the ancestral karyotype of supertribes Camelinodae and Brassicodae (ACBK). Four Chromosome-Like Synteny Blocks (CLSBs) were identified as protochromosomes shared by Camelinodae (Lineage I) and Brassicodae (Lineage II) genomes and labeled ACBK1 to ACBK4. Each colored box corresponds to one of the four protochromosomes inferred.

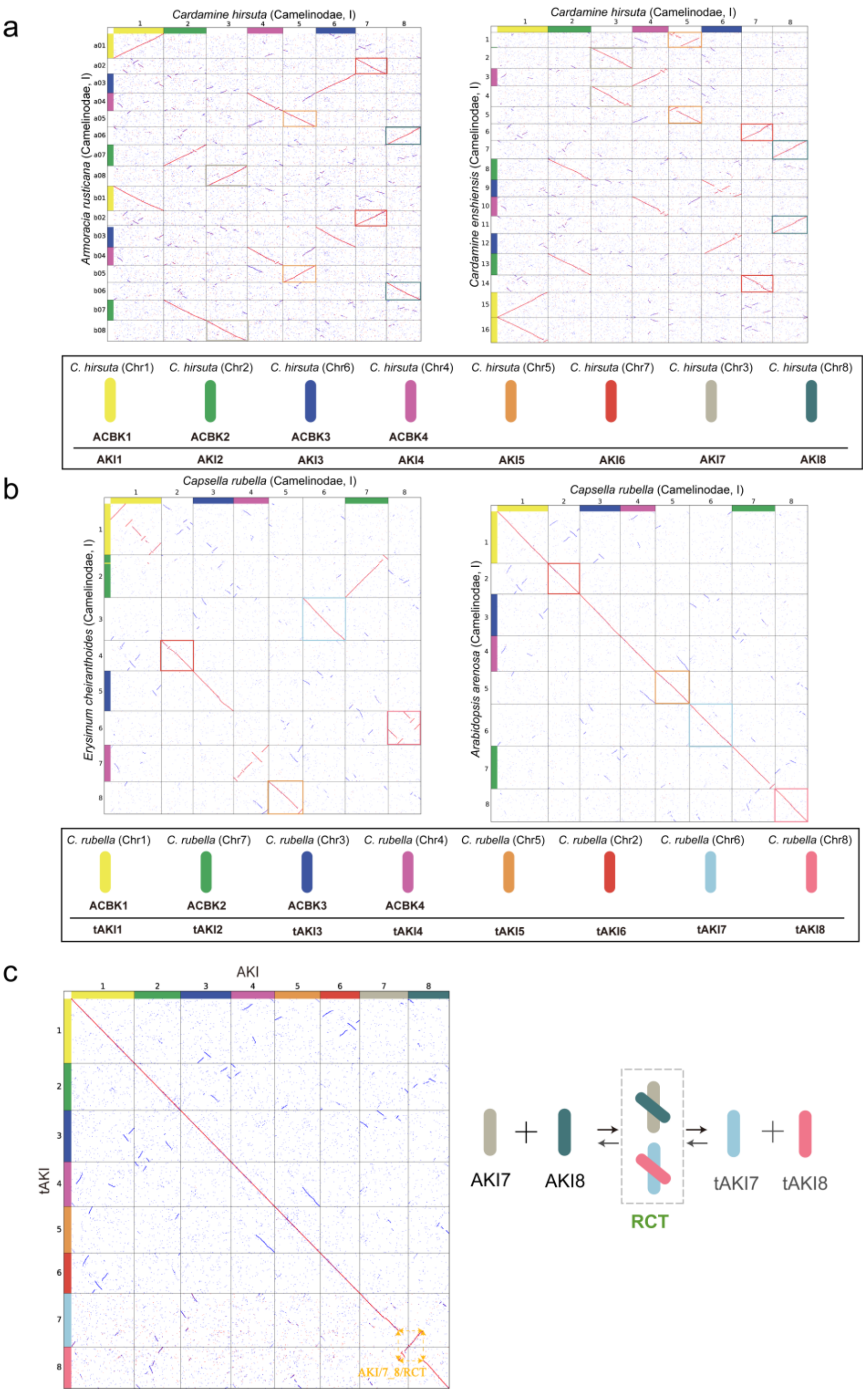

Supplementary Figure 3. Reconstruction of ancestral karyotype AKI and its translocation variant tAKI in Camelinodae. **(*a*)** The ancestral karyotype AKI comprises eight protochromosomes, including four newly identified ones (AKI5-8). **(*b*)** The ancestral karyotype tAKI also consists of eight protochromosomes, with four additional ones (tAKI5-8) identified. **(*c*)** Two identical protochromosomes (AKI5=tAKI5, AKI6=tAKI6) are shared by AKI and tAKI ancestral genomes, whereas a reciprocal translocation between chromosomes AKI7 and AKI8 differentiates AKI and tAKI genomes. As the reciprocal translocation AKI/7_8/RCT differentiating AKI and tAKI is a simple whole-arm translocation, it remains unclear which of the two genomes was closer to ACBK.

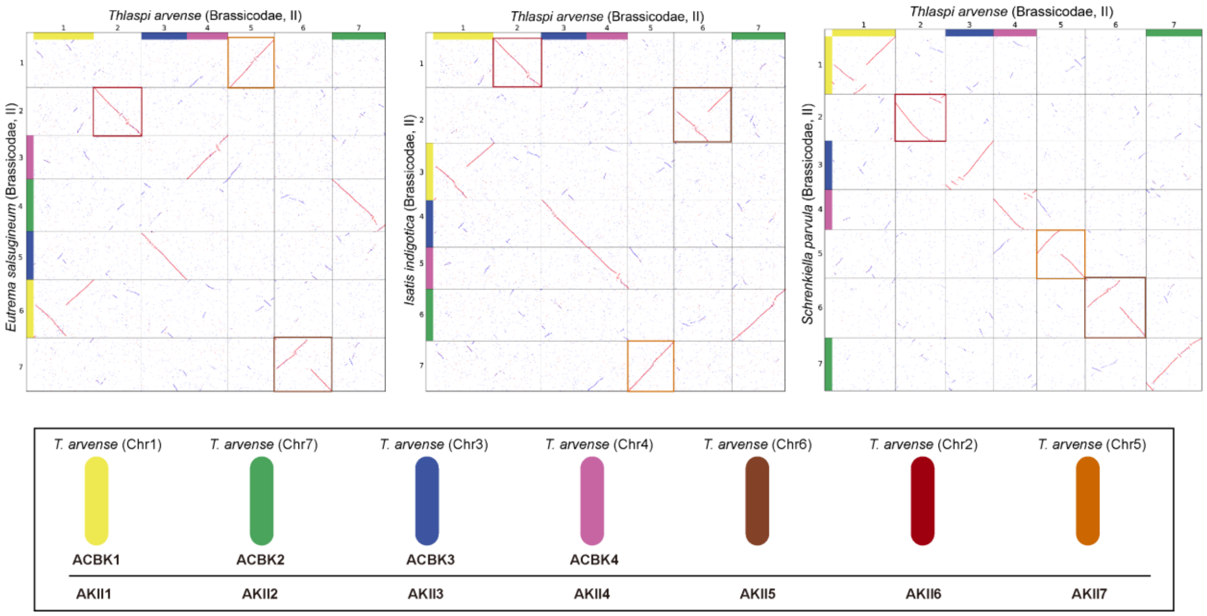

Supplementary Figure 4. Reconstruction of ancestral genome AKII in Brassicodae. The genome assemblies of species from tribes Eutremeae, Isatideae, Schrenkielleae, and Thlaspideae were used to infer the 7-chromosome AKII genome including three newly identified chromosomes (AKII5-7).

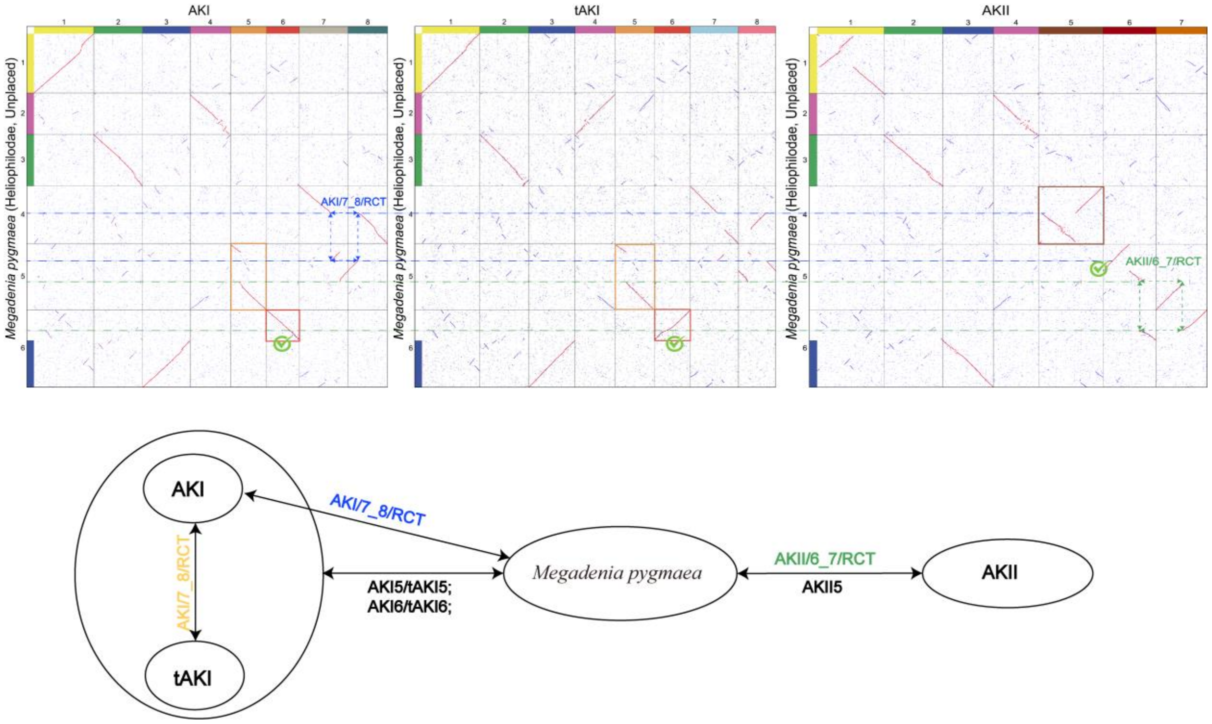

Supplementary Figure 5. Phylogenomic position of *Megadenia pygmaea* (Biscutelleae, Heliophilodae) between the supertribes Camelinodae and Brassicodae. Protochromosomes AKI5/tAKI5, AKI6/tAKI6 and AKII5 identified as CLSBs in the *M. pygmaea* genome confirm the phylogenetic position of the species/tribe between the Camelinodae and Brassicodae. Moreover, the fusion breakpoints of the reciprocal translocation lie within synteny blocks of *M. pygmaea* and AKII, indicative of a shared ancestral structure.

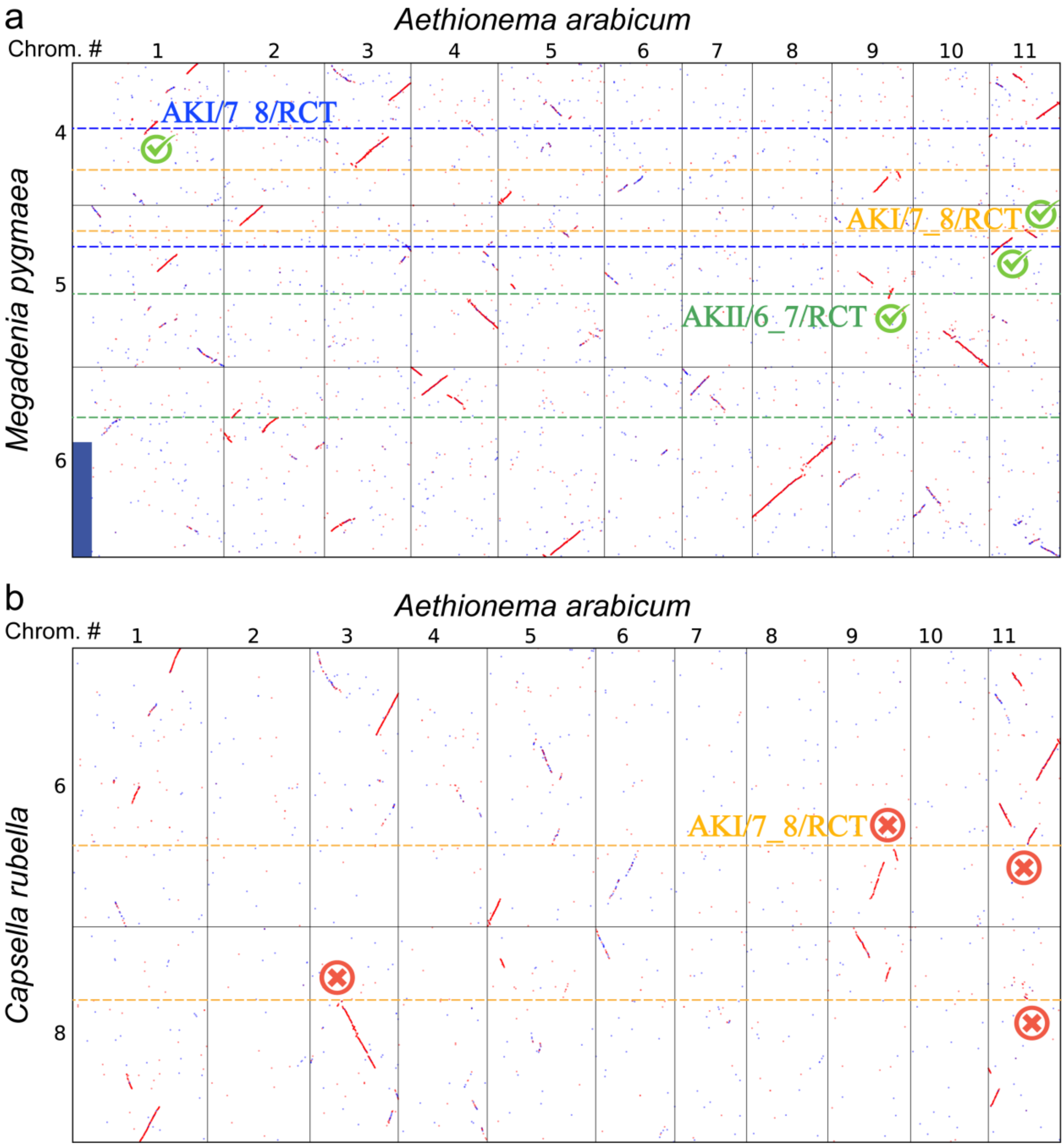

Supplementary Figure 6. Confirmation of the sequence of multiple reciprocal translocations using *Aethionema arabicum* as a reference. (*a*) *M. pygmaea* shares chromosomal rearrangements with *A. arabicum*. The blue AKI/7_8/RCT, yellow AKI/7_8/RCT, and green AKII/6_7/RCT respectively indicate translocations involving AKI with *M. pygmaea*, AKI with tAKI, and AKII with *M. pygmaea*. **(*b*)** The chromosome structures differ between *Capsella rubella* and *A. arabicum*, showing species-specific chromosomal structural variation in the former species. Green checkmarks indicate a support of the fusion events, while red crossmarks stand for a lack of support. We used the three species to verify the directionality of the three RCTs; similar conclusions can be obtained by replacing them with other species.

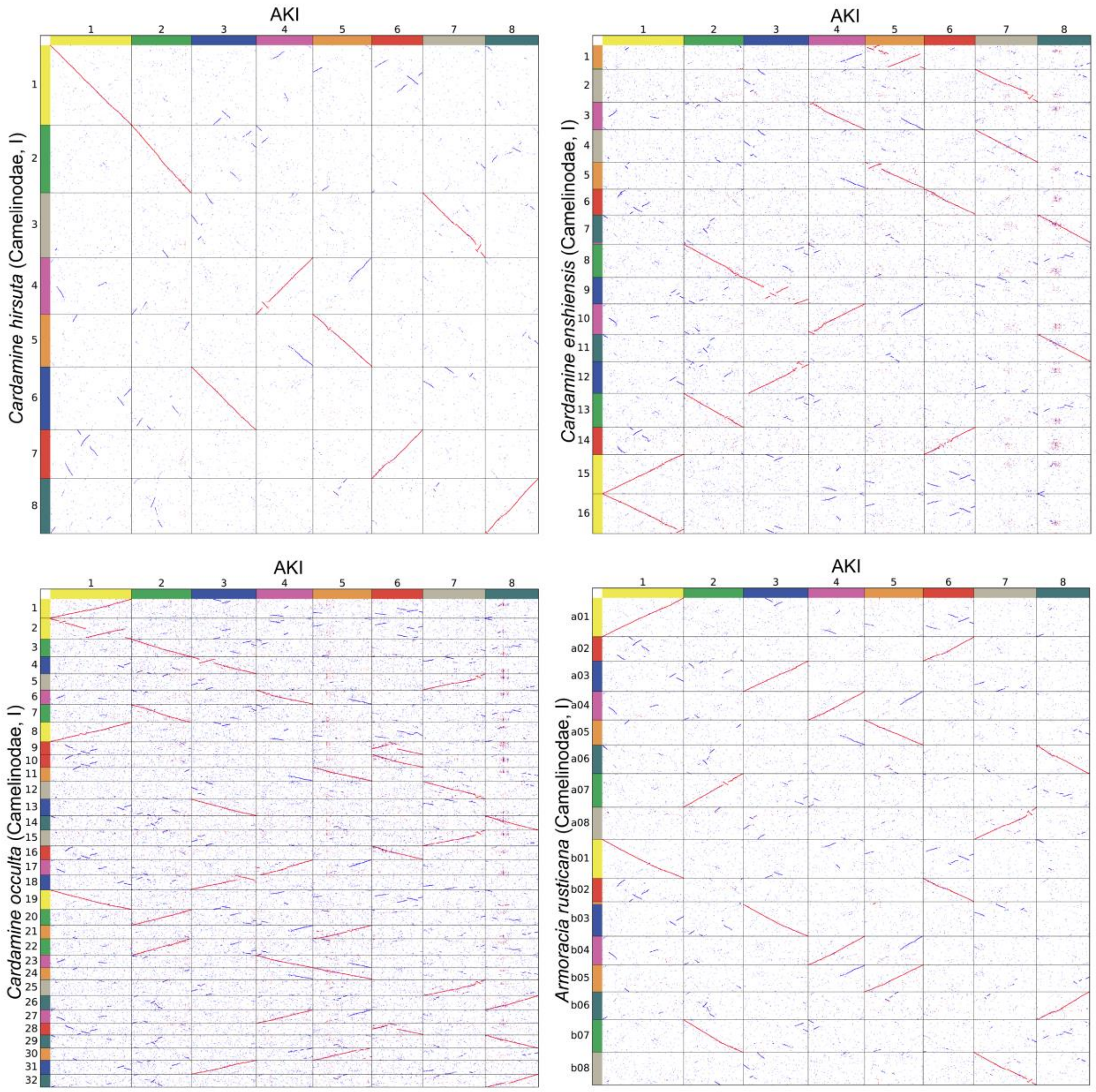

Supplementary Figure 7. Protochromosomes of the AKI ancestral genome are shared between four extant Cardamineae genomes.

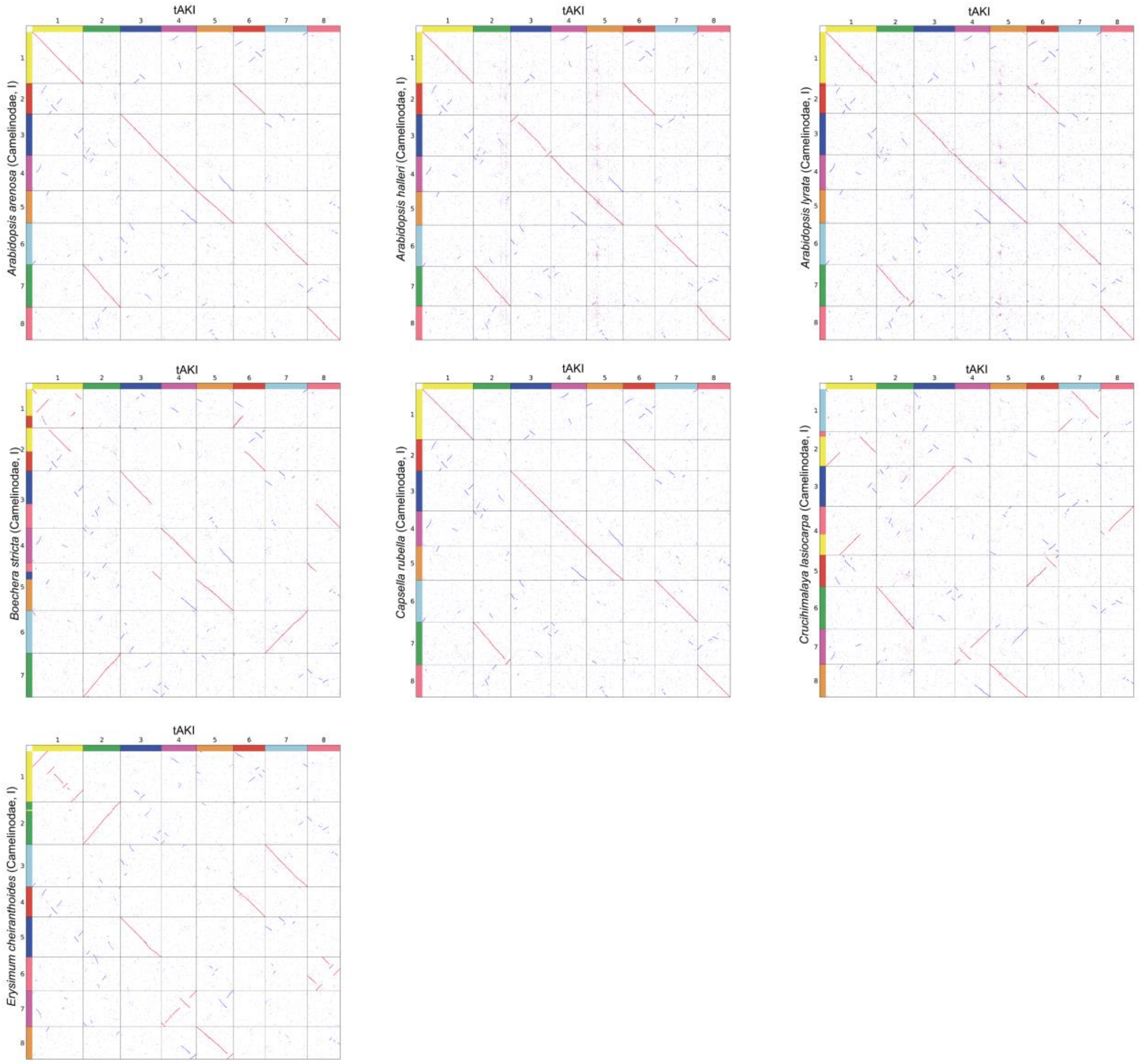

Supplementary Figure 8. Protochromosomes of tAKI are shared between genomes of five Camelinodae tribes (Arabidopsideae, Boechereae, Camelineae, Crucihimalayeae and Erysimeae).

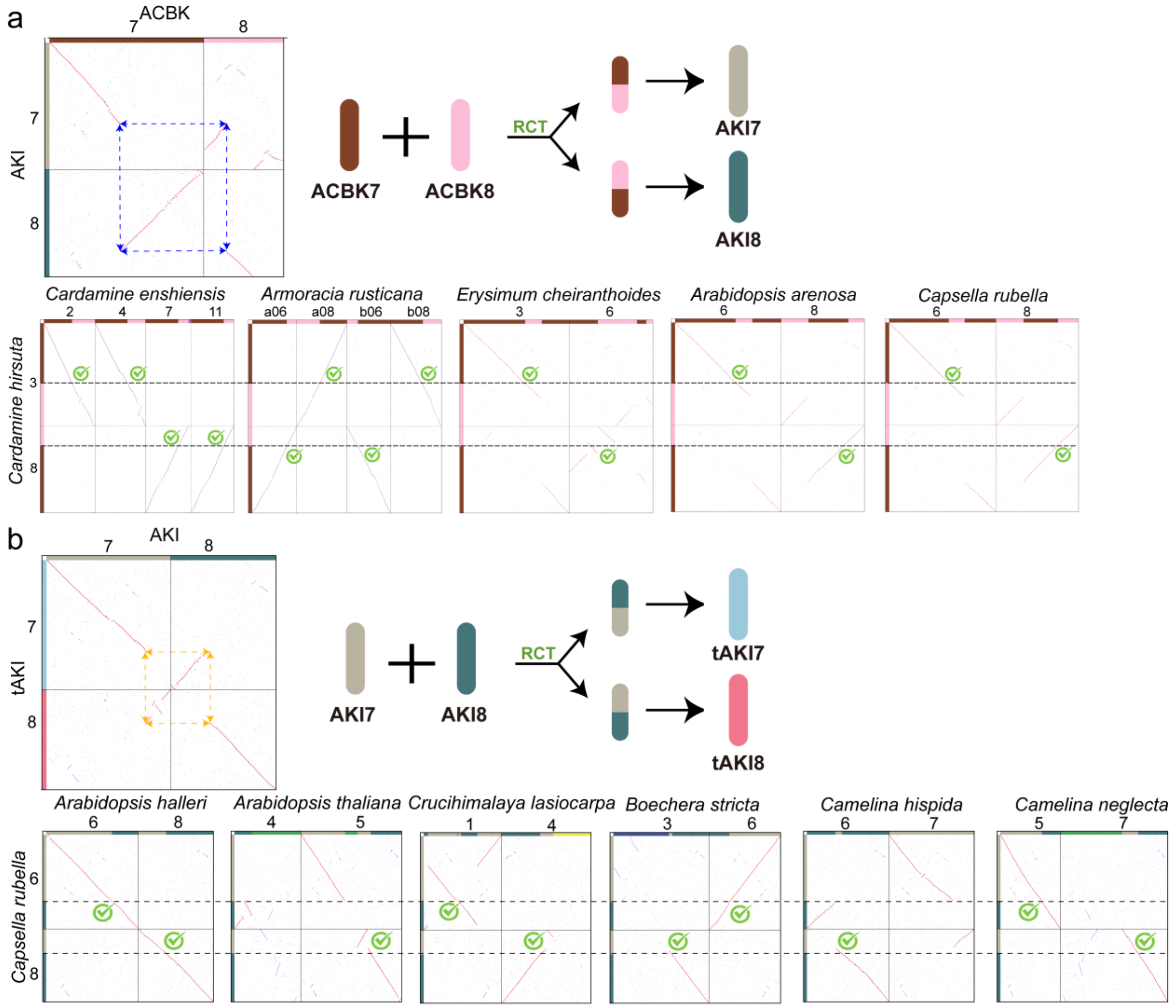

Supplementary Figure 9. Two phylogenetically informative reciprocal translocations in the Camelinodae. (*a*) The ACBK/7_8/RCT event is shared across Camelinodae tribes including the Cardamineae (*Armoracia rusticana*, *Cardamine hirsuta* and *C. enshiensis*). **(*b*)** The AKI/7_8/RCT event post-dates the divergence of the Cardamineae and the remaining Camelinodae tribes. RCT: reciprocal chromosome translocation. Green checkmarks indicate a support of the fusion events.

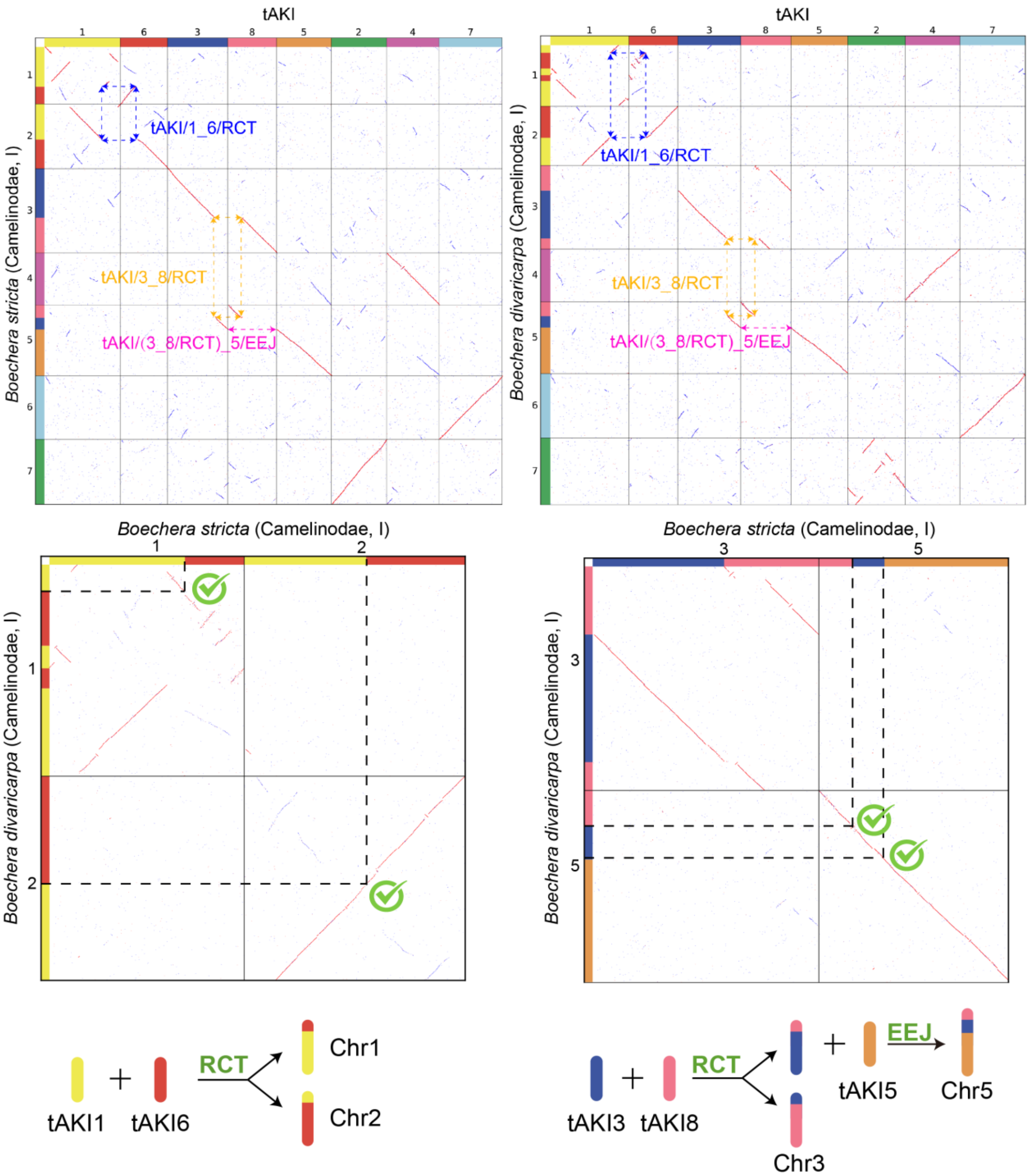

Supplementary Figure 10. Chromosomal rearrangements shared by *Boechera* species. Both *Boechera* species share signatures of two RCTs (tAKI/1_6/RCT, tAKI/3_8/RCT) and one EEJ (tAKI/(3_8/RCT)_5/EEJ). RCT: reciprocal chromosome translocation; EEJ: end-to-end joining. Green checkmarks indicate a support of the shared chromosomal rearrangements.

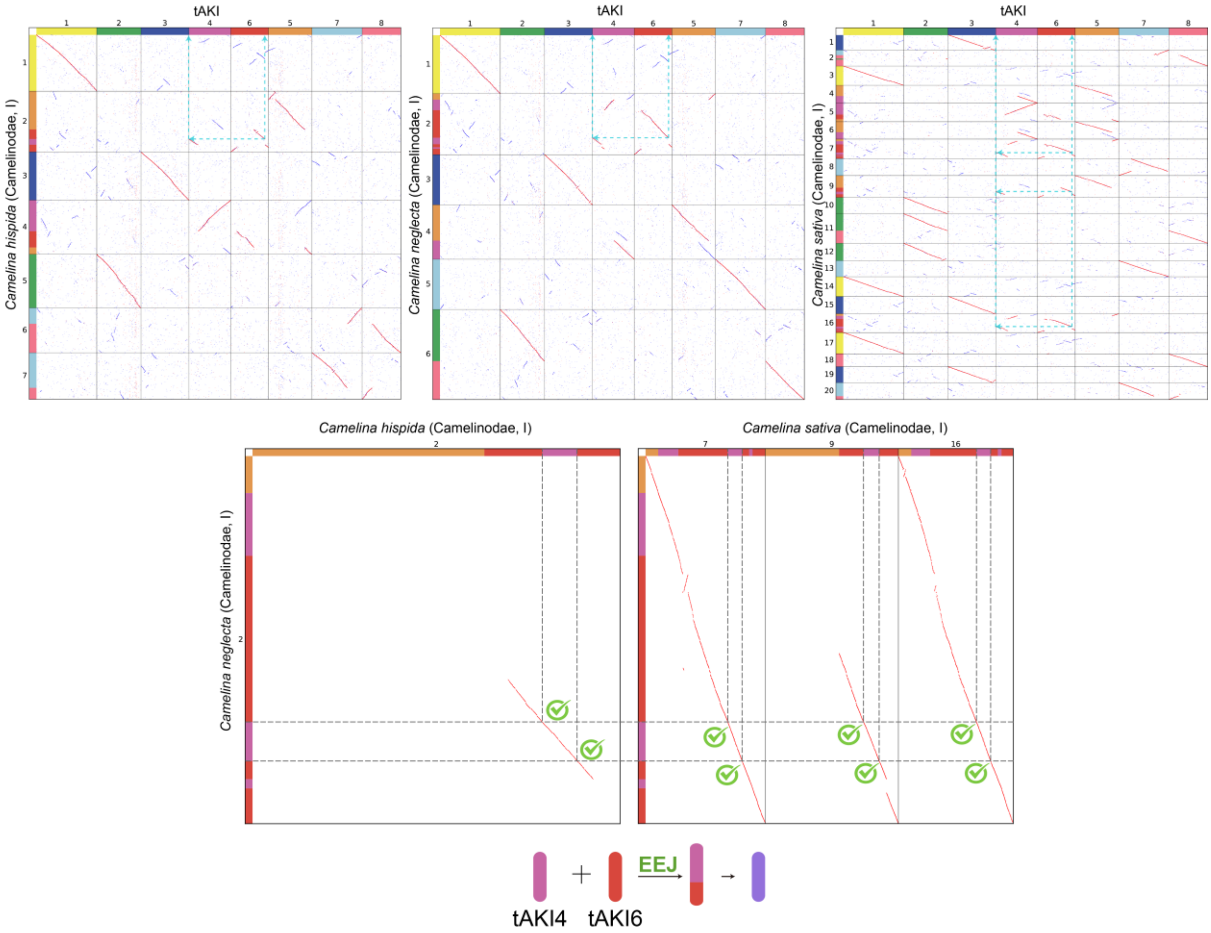

Supplementary Figure 11. A shared chromosome fusion in *Camelina*. *Camelina* species share a chromosomal rearrangement involving tAKI4 and tAKI6, likely resulting from a single EEJ event (tAKI/4_6/EEJ) as the two fusion breakpoints are within one syntenic block. EEJ: end-to-end joining. Green checkmarks indicate a support for the fusion events.

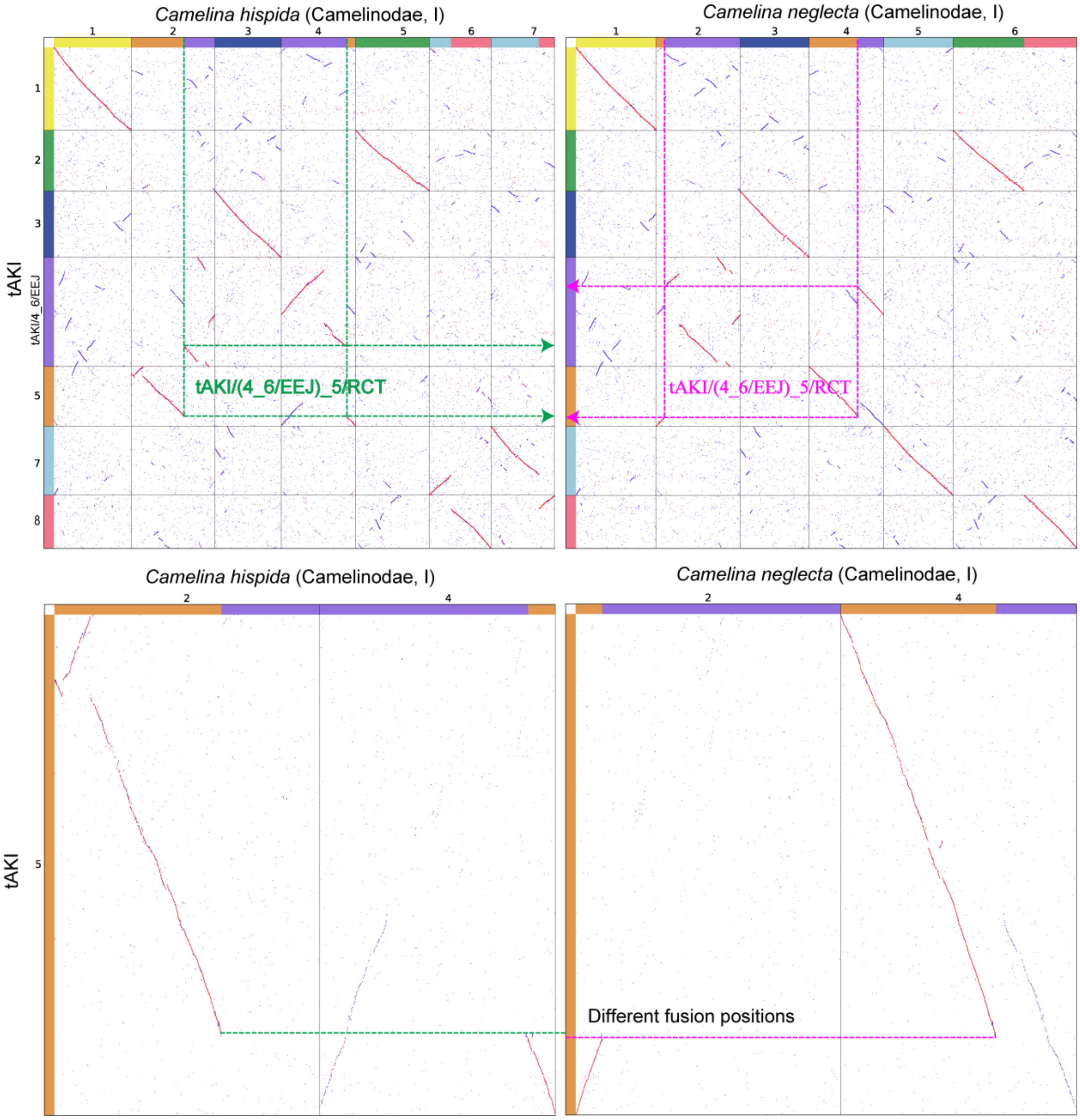

Supplementary Figure 12. Different fusion breakpoints on the same protochromosomes indicate the incidence of similar, but distinct chromosomal rearrangements. Both *Cardamine hispida* and *C. neglecta* have reciprocal translocations involving tAKI/4_6/EEJ and tAKI5, with different fusion breakpoints suggesting independent chromosomal rearrangements. RCT: reciprocal chromosome translocation.

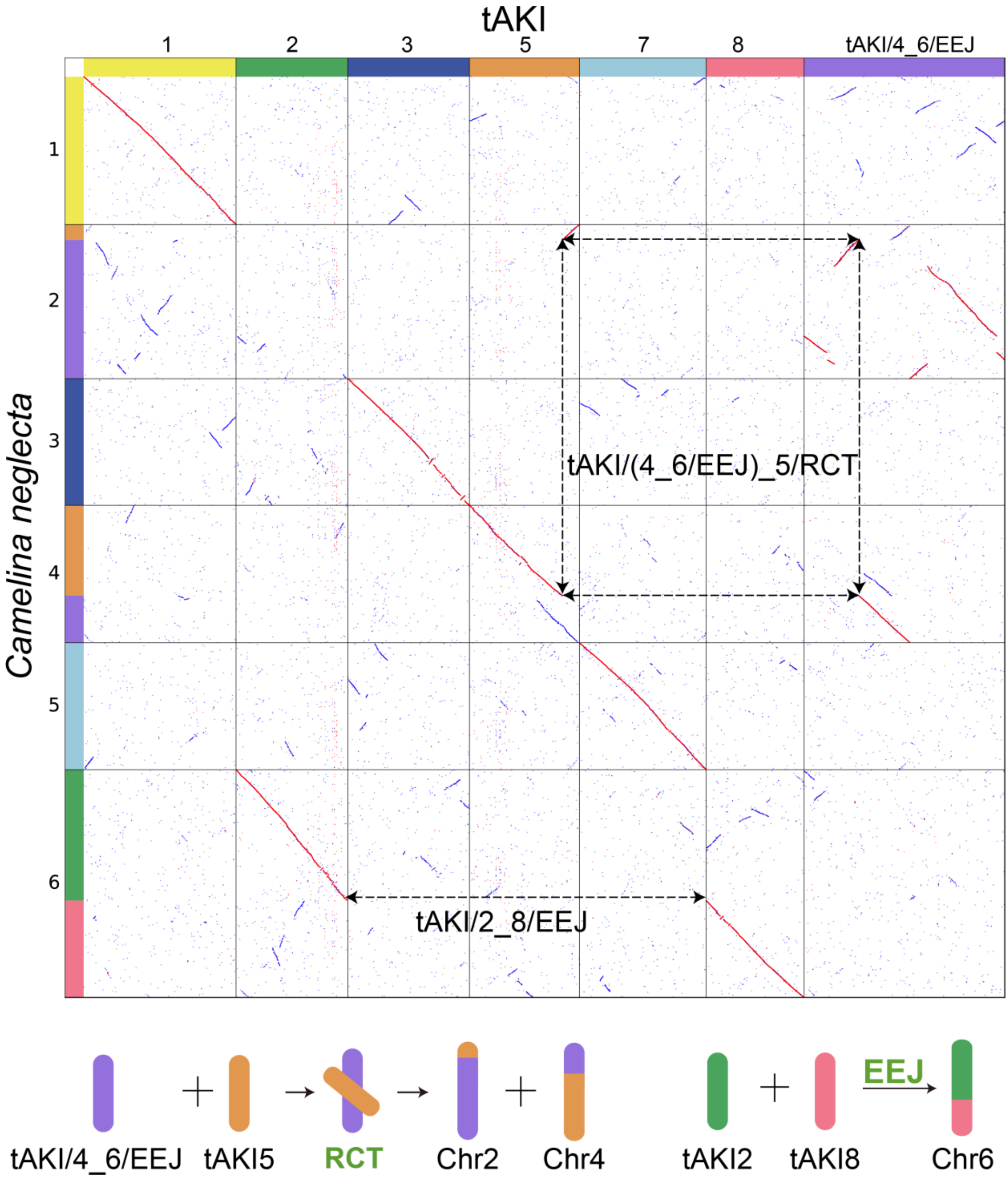

Supplementary Figure 13. Two additional chromosome fusion events in the evolution of the *Camelina neglecta* genome. RCT: reciprocal chromosome translocation; EEJ: end-to-end joining.

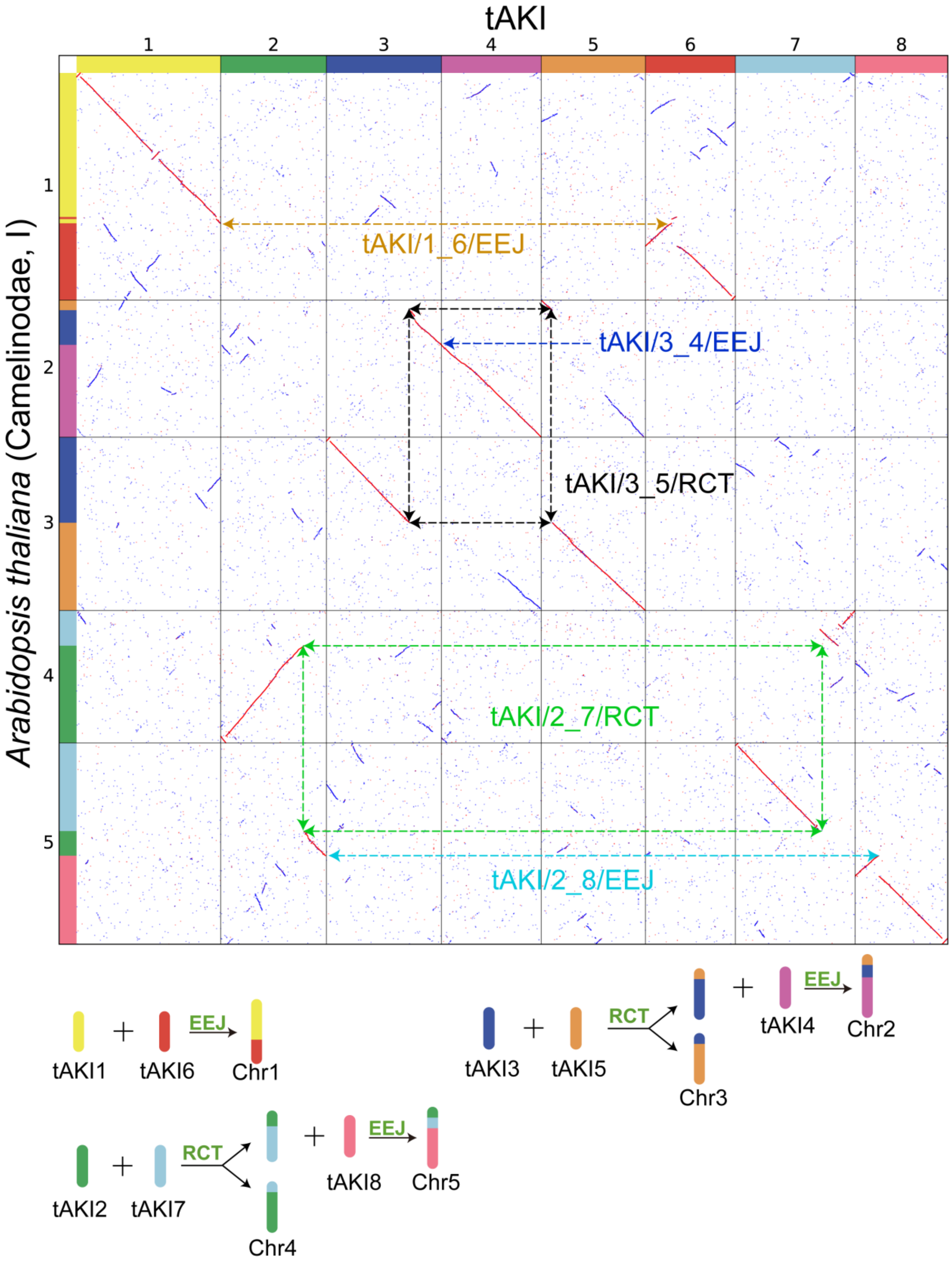

Supplementary Figure 14. The origin of five chromosomes of *Arabidopsis thaliana* (Arabidopsideae) from the eight chromosomes of tAKI. RCT: reciprocal chromosome translocation; EEJ: end-to-end joining.

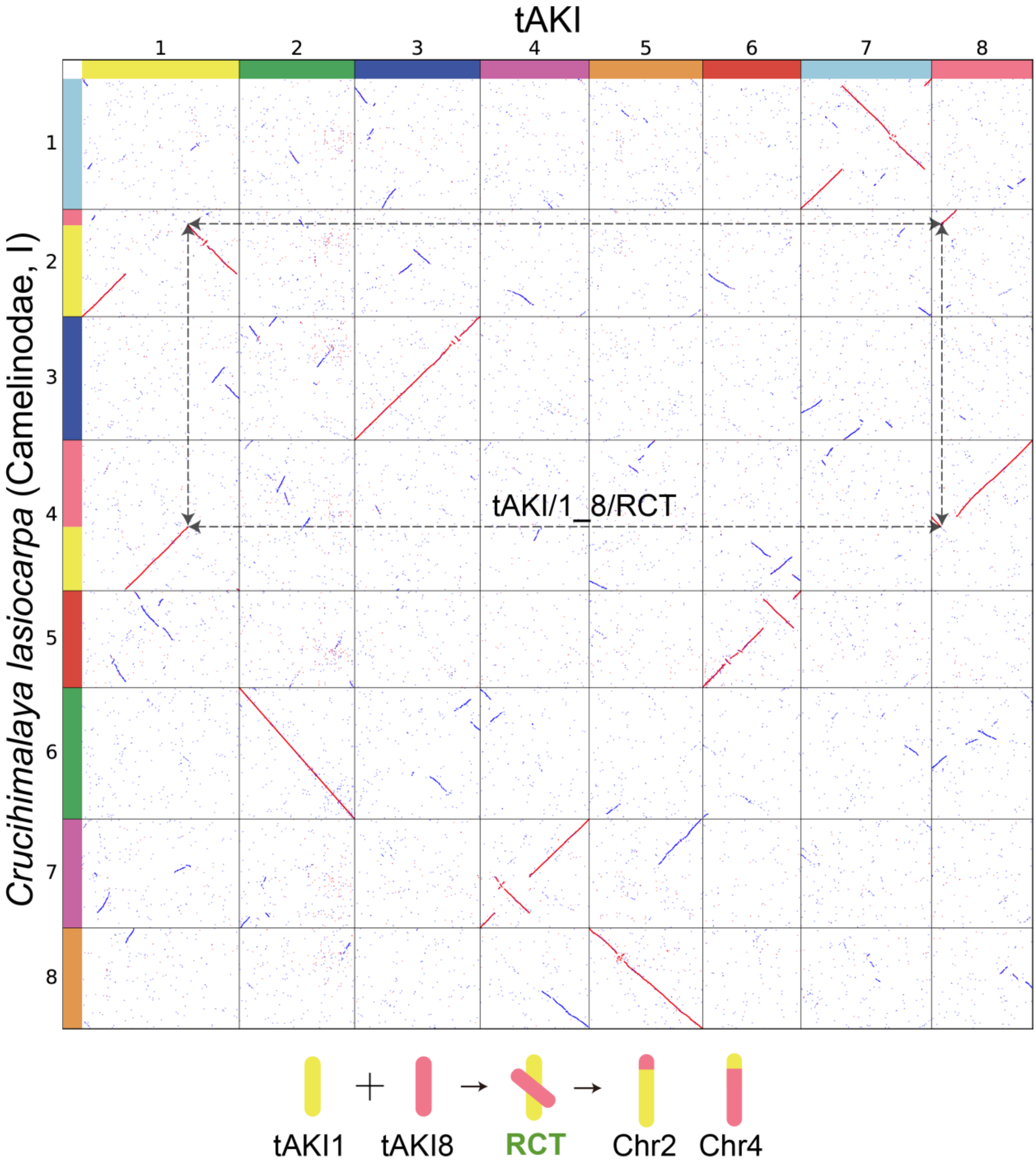

Supplementary Figure 15. The genome of *Crucihimalaya lasiocarpa* (Crucihimalayeae) compared with the ancestral tAKI genome. RCT: reciprocal chromosome translocation.

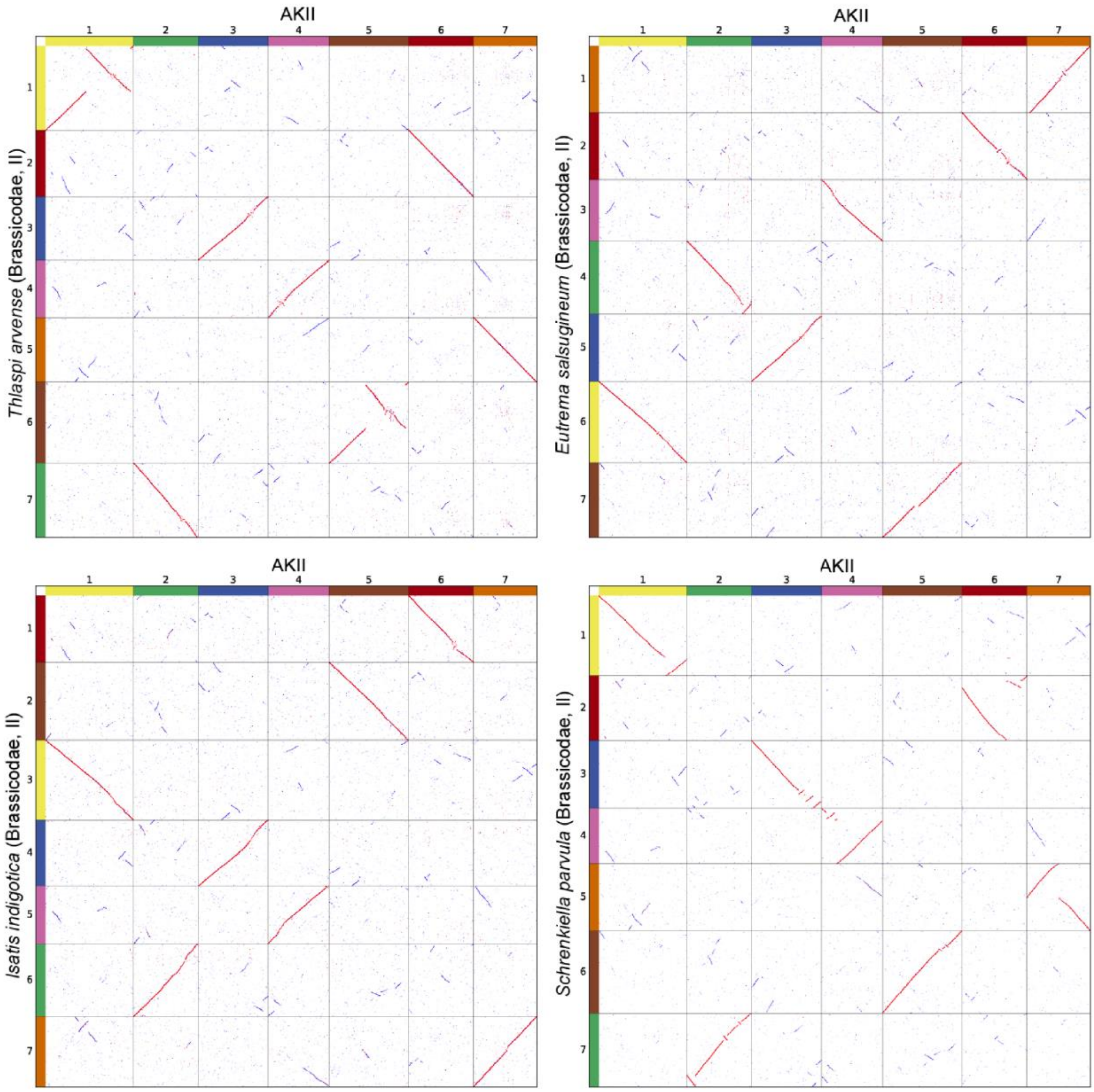

Supplementary Figure 16. All protochromosomes of AKII retained their ancestral structure in the analyzed genomes of four Brassicodae tribes (Eutremeae, Isatideae, Schrenkielleae and Thlaspideae).

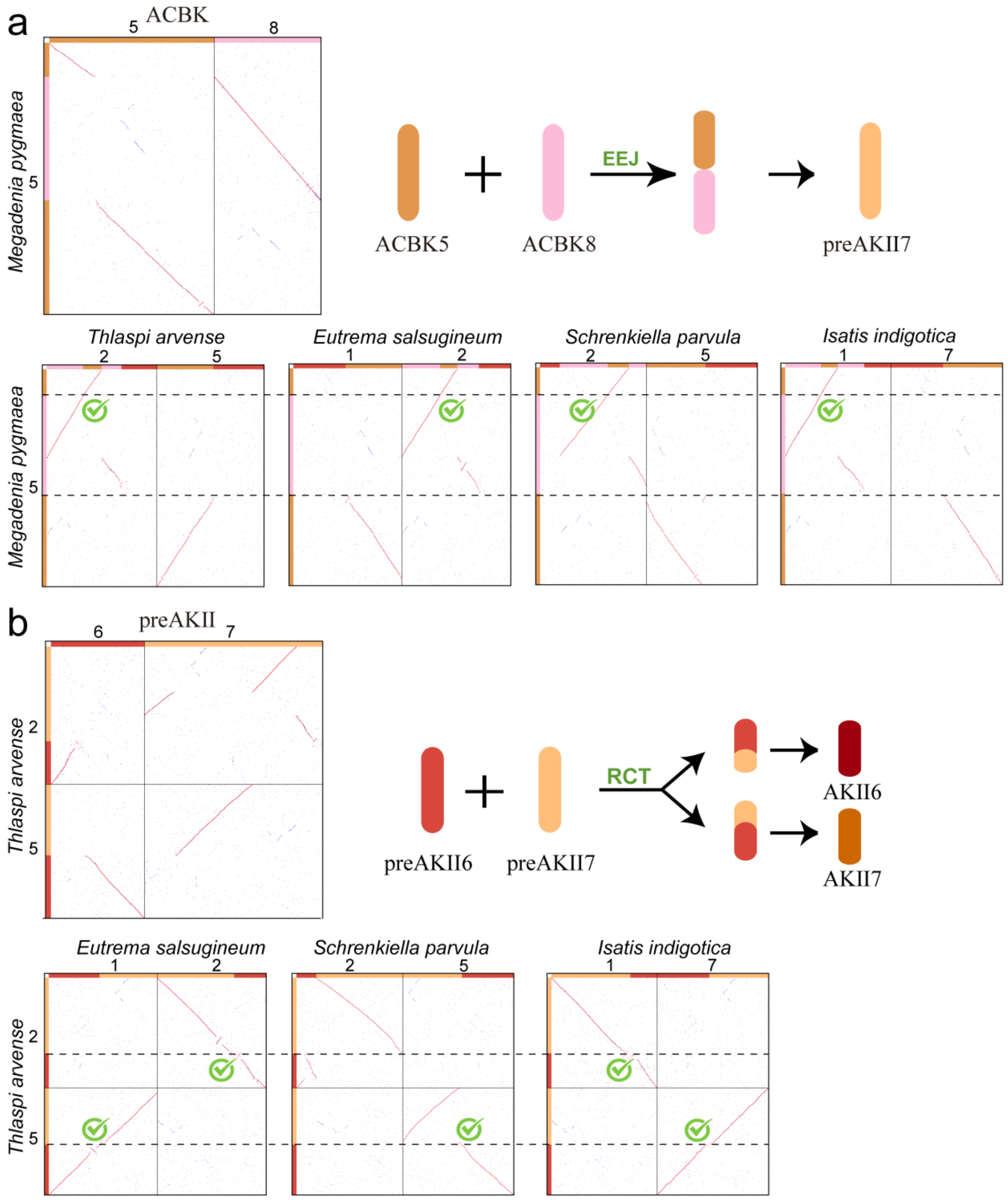

Supplementary Figure 17. Two shared fusion events in Brassicodae tribes. (*a*) The ACBK/5_8/EEJ event is shared between the Brassicodae genomes as well as between the Brassicodae and Heliophilodae (*Megadenia pygmaea*) genomes. **(*b*)** The preAKII/6_7/RCT event is shared between Brassicodae genomes. RCT: reciprocal chromosome translocation; EEJ: end-to-end joining. Green checkmarks indicate a support of the fusion events.

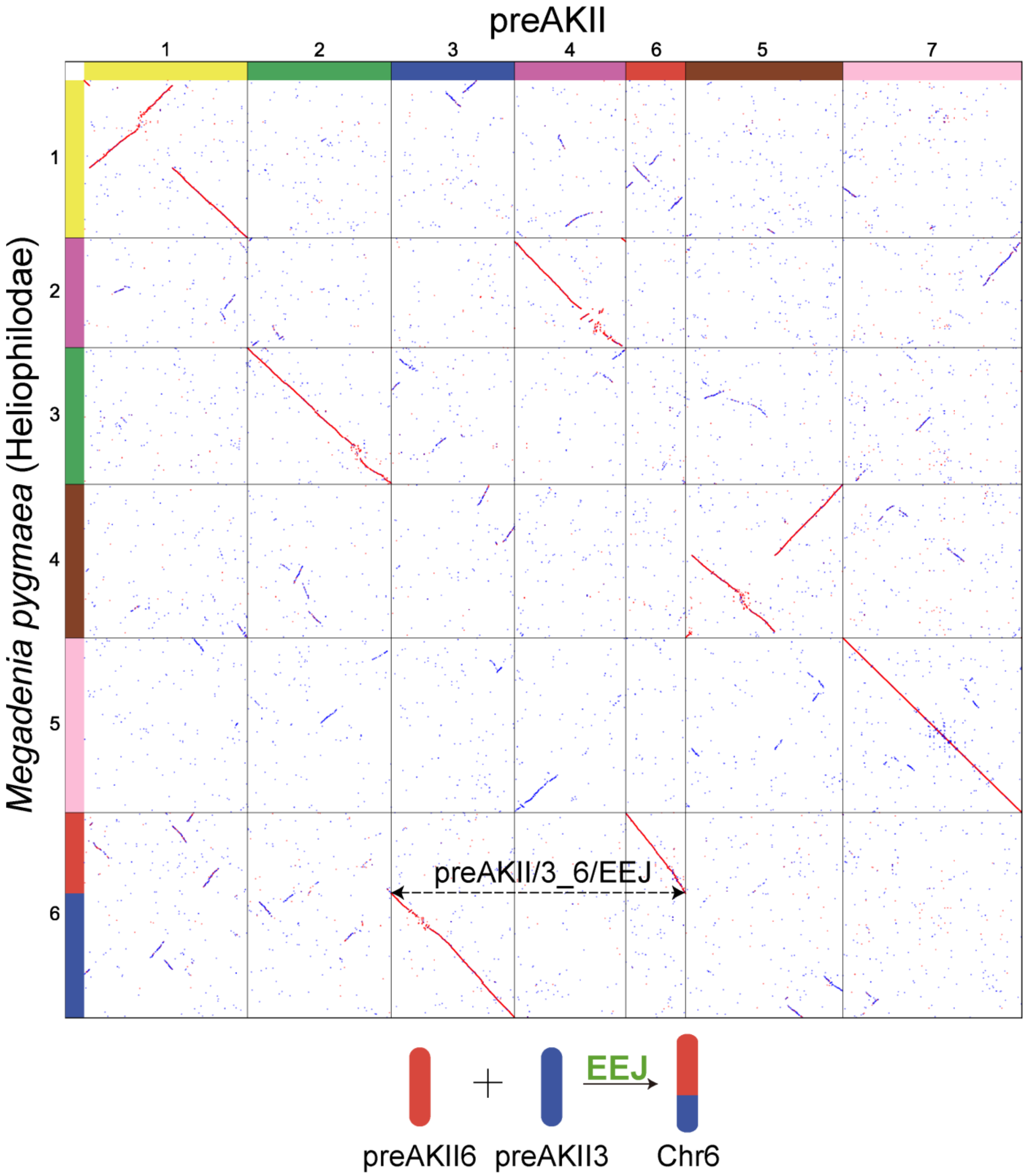

Supplementary Figure 18. The origin of the 6-chromosome genome of *Megadenia pygmaea*. Chromosome 6 was formed by an EEJ between protochromosomes preAKII6 and preAKII3. EEJ: end-to-end joining.

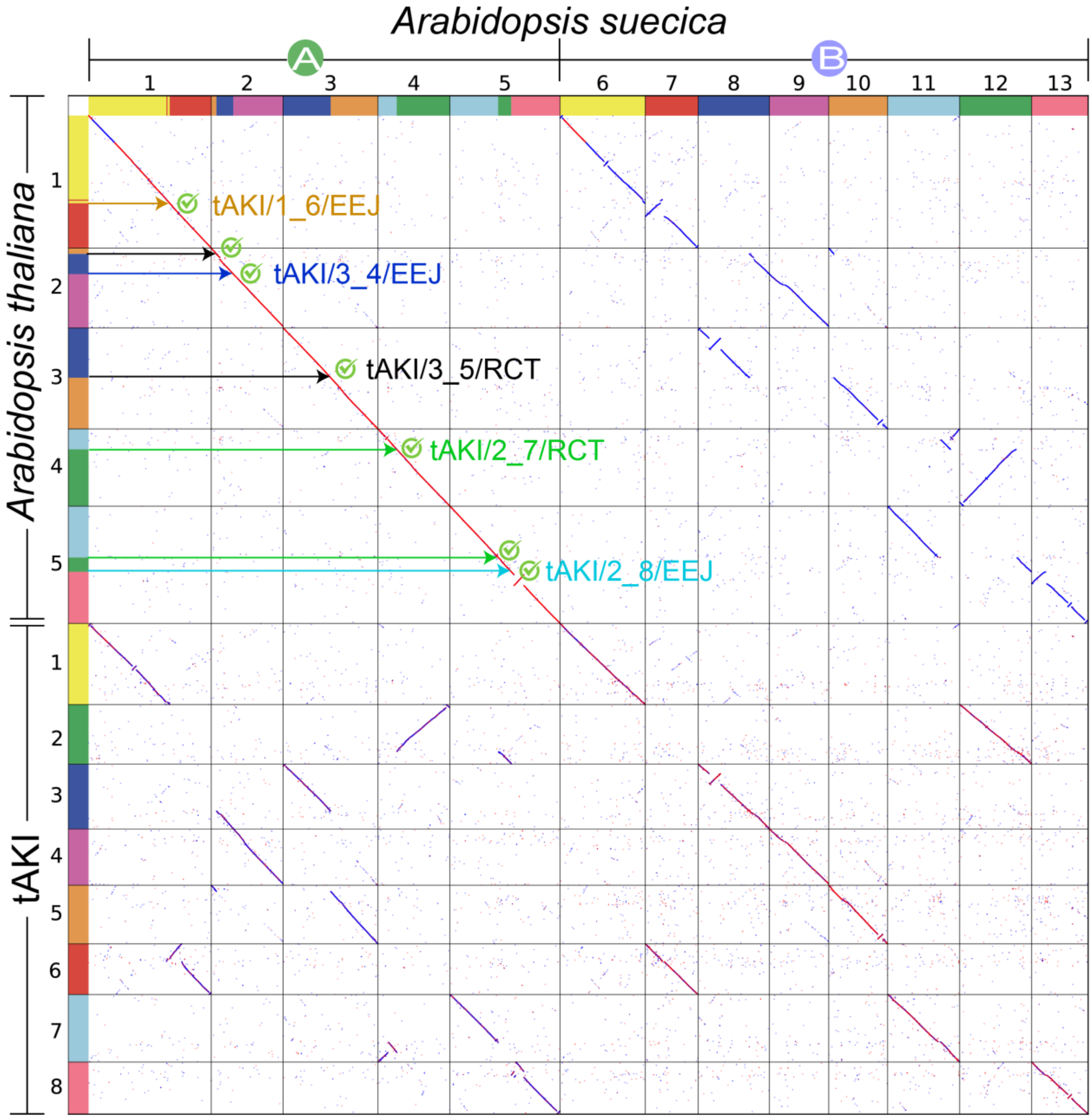

Supplementary Figure 19. Chromosome fusion events in the allotetraploid *Arabidopsis suecica* (*n* = 13). *A. suecica* has all specific chromosome fusions found in *A. thaliana* (subgenome A, *n* = 5); subgenome B (*n* = 8) was contributed by *A. arenosa*. RCT: reciprocal chromosome translocation; EEJ: end-to-end joining. Green checkmarks indicate collinearity support for the fusion events.

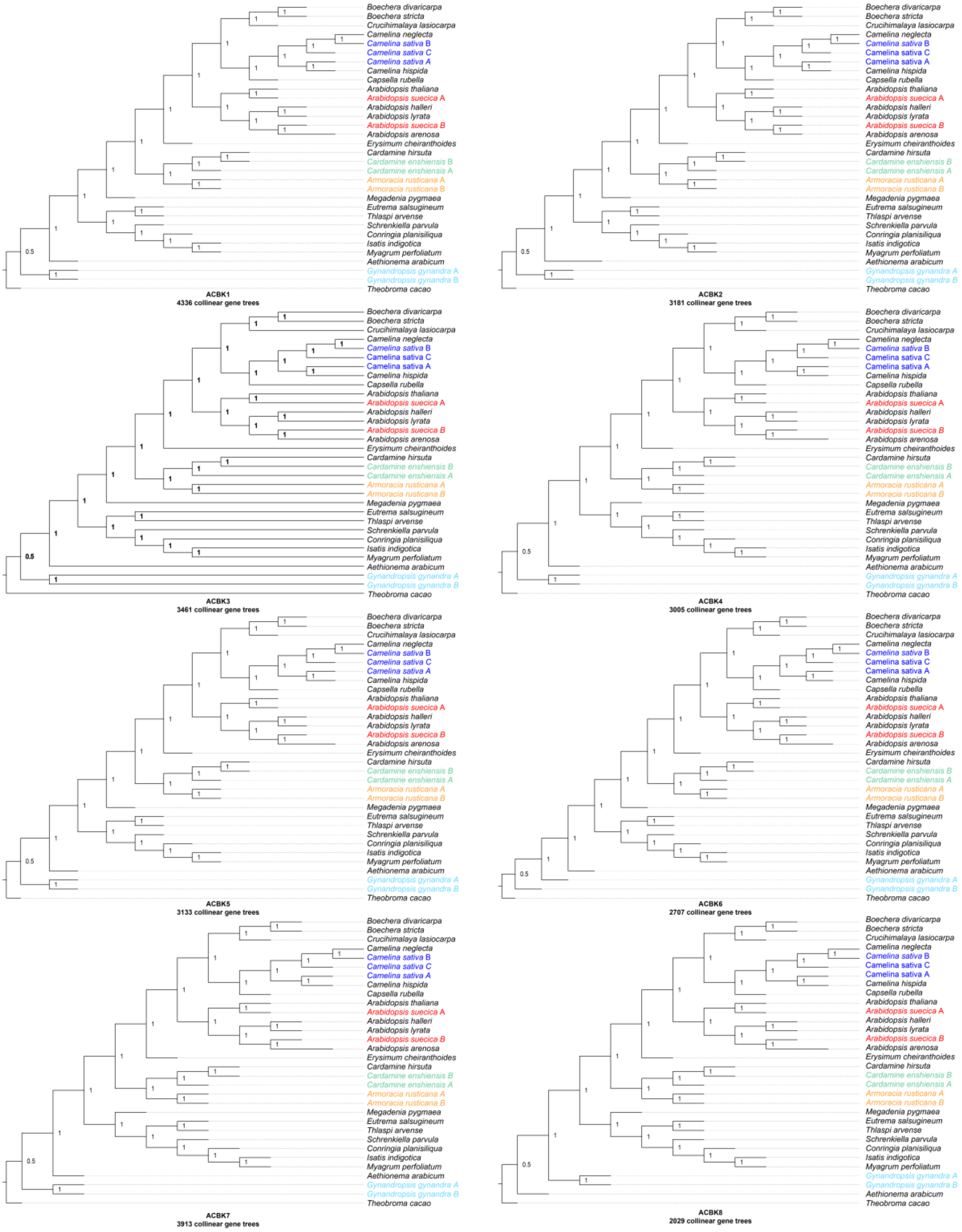

Supplementary Figure 20. Phylogenetic relationships using collinear genes extracted from each of the 8 ancestral chromosomes of ACBK genome shared by the supertribes Camelinodae and Brassicodae. Each subgenome tree was inferred from the number of collinear genes (after lineage-specific polyploidization events) given below the respective tree with ASTER (https://github.com/chaoszhang/ASTER). Numbers at nodes represent posterior probabilities. If the subgenomes of a species do not cluster on one branch but are mixed with other species, an allopolyploid origin of the former species is inferred (e.g. *Arabidopsis suecica*, *Camelina sativa*, and *Cardamine enshiensis*). Source data are provided as a Source Data file.

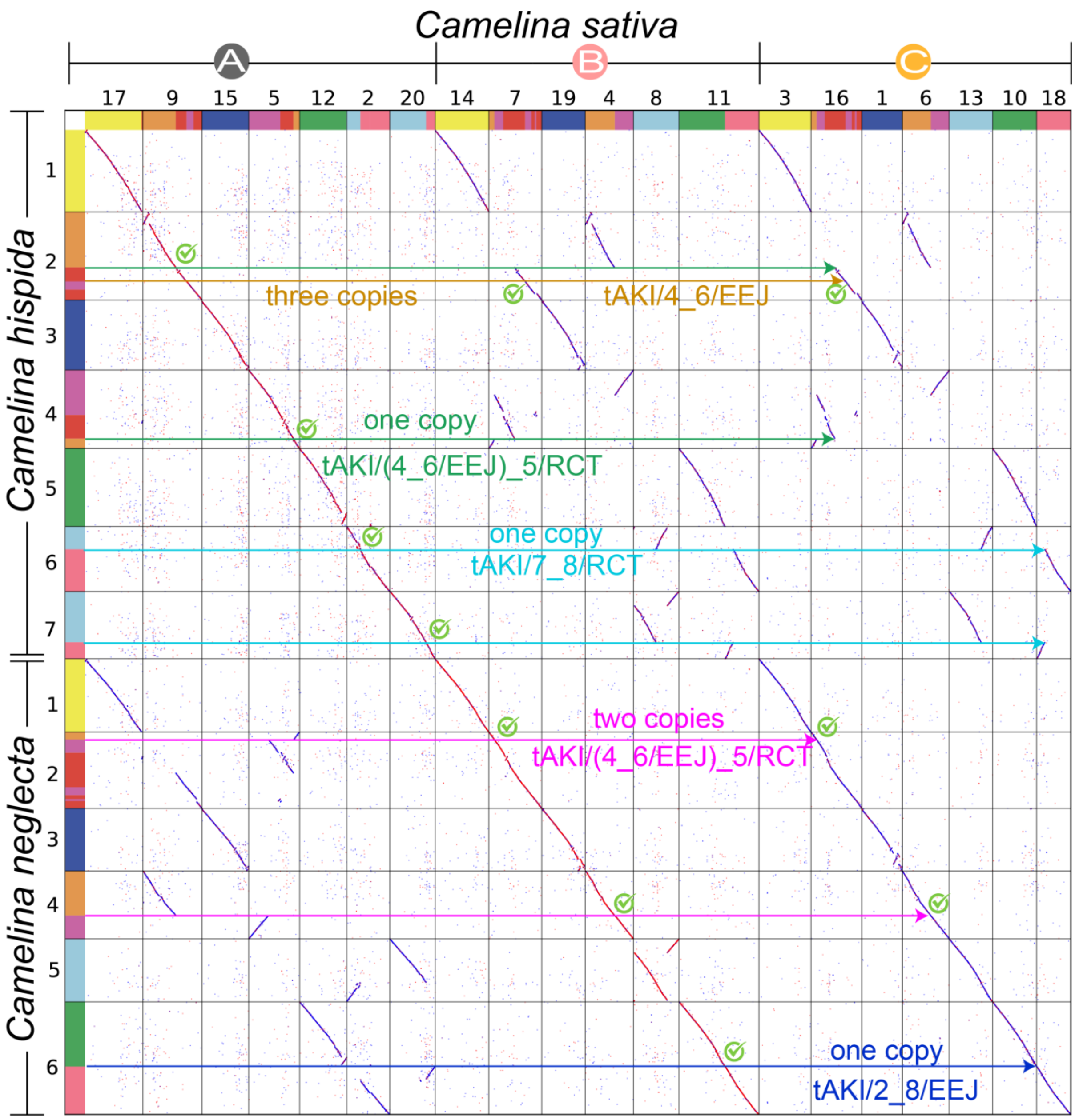

Supplementary Figure 21. Chromosome fusion events in *Camelina sativa* and its subgenome phasing. Based on the dot plot between *C. hispida* (n = 7), *C. neglecta* (n = 6) and *C. sativa* (n = 20), the distribution of best homologous genes (red dots) and shared fusion breakpoints indicate that hexaploid *C. sativa* genome can be divided into three subgenomes: A, B and C. Subgenome C shares the tAKI/(4_6/EEJ)_5/RCT event with *C. neglecta* but lacks the tAKI/2_8/EEJ, indicative of a closer evolutionary relationship with *C. neglecta*. RCT: reciprocal chromosome translocation; EEJ: end-to-end joining. Green checkmarks indicate collinearity support for the fusion events.

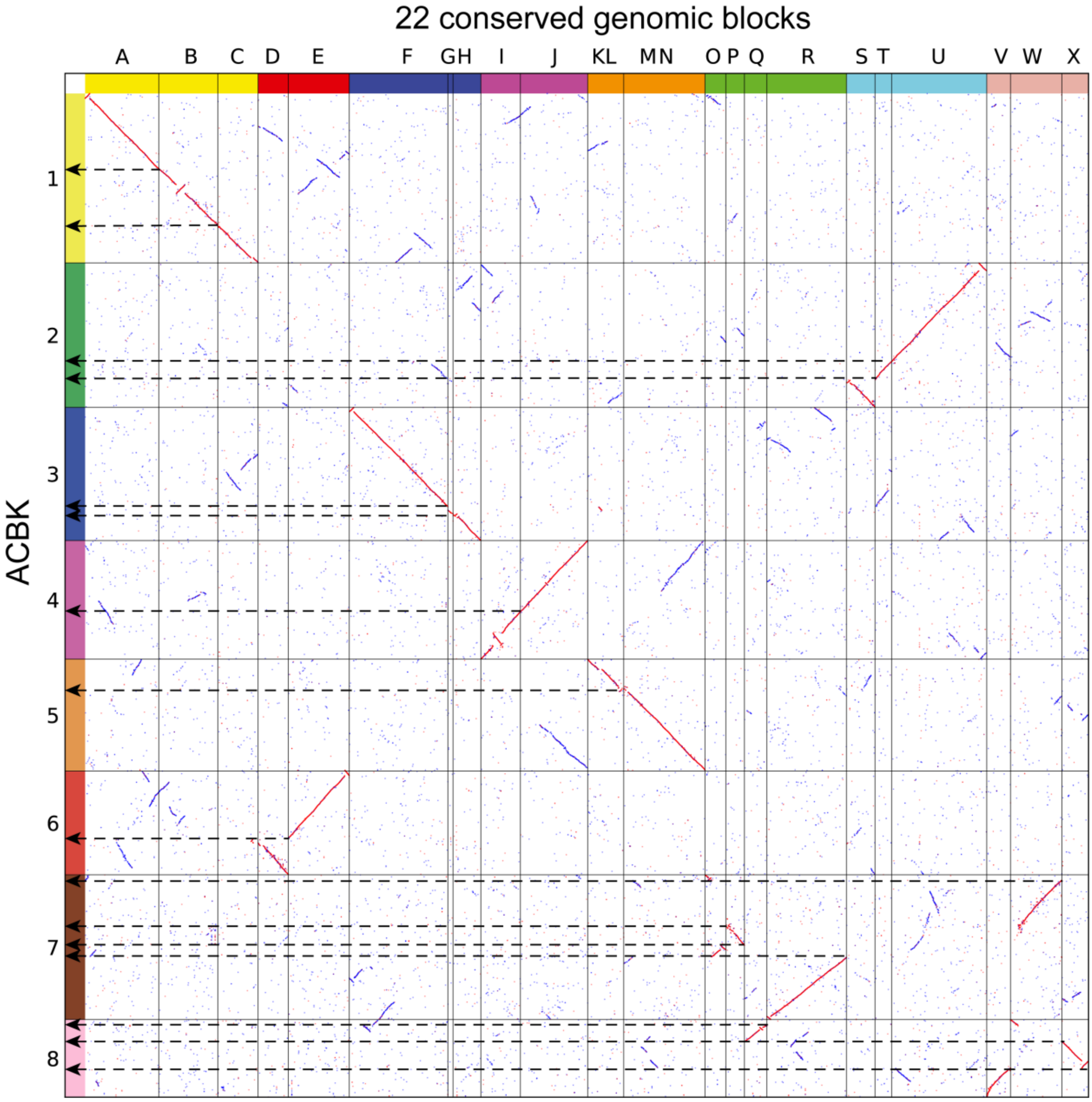

Supplementary Figure 22. The corresponding positions of the 22 conserved genomic blocks boundaries on the eight protochromosomes of ACBK.

Supplementary reference

1. Mandáková, T., Marhold, K. & Lysak, M. A. The widespread crucifer species *Cardamine flexuosa* is an allotetraploid with a conserved subgenomic structure. *New Phytol.* **201**, 982-992 (2014).
