# Supplementary material for: Chromosome fusions shaped karyotype evolution and evolutionary relationships in the model family Brassicaceae
Source: Nat Commun. 2025 May 19;16:4631. doi: 10.1038/s41467-025-59640-2 (PMC12089291; doi:10.1038/s41467-025-59640-2)
Supplement: Supplementary file 3 — Description of Additional Supplementary Files [file 41467_2025_59640_MOESM3_ESM.pdf]

**Description of Additional Supplementary Files**

File Name: Supplementary Data 1
Description: Statistics of data sources used in this study.

File Name: Supplementary Data 2
Description: Detailed locations of fusion breakpoints for shared major chromosomal rearrangements across sample species.

File Name: Supplementary Data 3
Description: The location of the fusion position on the ancestral chromosome.
