# Supplementary material for: Chromosome fusions shaped karyotype evolution and evolutionary relationships in the model family Brassicaceae
Source: Nat Commun. 2025 May 19;16:4631. doi: 10.1038/s41467-025-59640-2 (PMC12089291; doi:10.1038/s41467-025-59640-2)
Supplement: Supplementary file 7 — Reporting Summary [file 41467_2025_59640_MOESM7_ESM.pdf]

nature portfolio

Corresponding author(s): Martin A. Lysak

Last updated by author(s): Apr 10, 2025

Reporting Summary

Nature Portfolio wishes to improve the reproducibility of the work that we publish. This form provides structure for consistency and transparency in reporting. For further information on Nature Portfolio policies, see our Editorial Policies and the Editorial Policy Checklist.

Statistics

For all statistical analyses, confirm that the following items are present in the figure legend, table legend, main text, or Methods section.

n/a	Confirmed	
☐	☒	The exact sample size (*n*) for each experimental group/condition, given as a discrete number and unit of measurement
☐	☒	A statement on whether measurements were taken from distinct samples or whether the same sample was measured repeatedly
☒	☐	The statistical test(s) used AND whether they are one- or two-sided<br>*Only common tests should be described solely by name; describe more complex techniques in the Methods section.*
☒	☐	A description of all covariates tested
☒	☐	A description of any assumptions or corrections, such as tests of normality and adjustment for multiple comparisons
☒	☐	A full description of the statistical parameters including central tendency (e.g. means) or other basic estimates (e.g. regression coefficient) AND variation (e.g. standard deviation) or associated estimates of uncertainty (e.g. confidence intervals)
☒	☐	For null hypothesis testing, the test statistic (e.g. *F*, *t*, *r*) with confidence intervals, effect sizes, degrees of freedom and *P* value noted<br>*Give P values as exact values whenever suitable.*
☒	☐	For Bayesian analysis, information on the choice of priors and Markov chain Monte Carlo settings
☒	☐	For hierarchical and complex designs, identification of the appropriate level for tests and full reporting of outcomes
☒	☐	Estimates of effect sizes (e.g. Cohen's *d*, Pearson's *r*), indicating how they were calculated

Our web collection on statistics for biologists contains articles on many of the points above.

Software and code

Policy information about availability of computer code

Data collection	No software was used to collect the data.
Data analysis	WGDI v0.74, ASTER v1.19, MUSCLE v5.3, MAFFT v7.526, IQ-TREE v2.3.6, PAML v4.10.7, PAL2NAL v14, Liftoff v1.6.3, DIAMOND v2.1.10.164.

For manuscripts utilizing custom algorithms or software that are central to the research but not yet described in published literature, software must be made available to editors and reviewers. We strongly encourage code deposition in a community repository (e.g. GitHub). See the Nature Portfolio guidelines for submitting code & software for further information.

Data

Policy information about availability of data

All manuscripts must include a data availability statement. This statement should provide the following information, where applicable:

- Accession codes, unique identifiers, or web links for publicly available datasets
- A description of any restrictions on data availability
- For clinical datasets or third party data, please ensure that the statement adheres to our policy

Ancestral karyotypes and phylogenetic data have been deposited in GitHub (https://github.com/SunPengChuan/Ancestral_Brassicaceae_Karyotype).

Research involving human participants, their data, or biological material

Policy information about studies with human participants or human data. See also policy information about sex, gender (identity/presentation), and sexual orientation and race, ethnicity and racism.

Reporting on sex and gender	No human participants or human data used in this study
Reporting on race, ethnicity, or other socially relevant groupings	No human participants or human data used in this study
Population characteristics	No human participants or human data used in this study
Recruitment	No human participants or human data used in this study
Ethics oversight	No human participants or human data used in this study

Note that full information on the approval of the study protocol must also be provided in the manuscript.

Field-specific reporting

Please select the one below that is the best fit for your research. If you are not sure, read the appropriate sections before making your selection.

- [x] Life sciences
- [ ] Behavioural & social sciences
- [ ] Ecological, evolutionary & environmental sciences

For a reference copy of the document with all sections, see nature.com/documents/nr-reporting-summary-flat.pdf

Life sciences study design

All studies must disclose on these points even when the disclosure is negative.

Sample size	The analyses performed in this manuscript are based on a sample size of 26 genomes from two Brassicaceae supertribes: Camelinodae (Lineage I) and Brassicodae (Lineage II), along with 7 genomes from other Brassicaceae lineages and one outgroup genome from Cleomaceae. Sample includes as many qualified chromosome-level genomes as possible from target clades, maximizing taxonomic coverage and following comparative genomics best practices to ensure reliable results.
Data exclusions	No data were excluded from the analyses.
Replication	All results can be reproduced 100% independently. Analyzing multiple genomic datasets from different species showed consistent ancestral karyotype evolution, and the inferred phylogenetic relationships matched previous studies, validating our findings.
Randomization	Randomization is irrelevant to this study, as we selected genomes from Brassicaceae supertribes Camelinodae (Lineage I) and Brassicodae (Lineage II). All genomes consistently demonstrated the stable inheritance of chromosomal fusions.
Blinding	Blinding is not relevant in this study. Since it focuses on genomic data analysis, there is no concern of subjective bias due to knowledge of group assignments.

Reporting for specific materials, systems and methods

We require information from authors about some types of materials, experimental systems and methods used in many studies. Here, indicate whether each material, system or method listed is relevant to your study. If you are not sure if a list item applies to your research, read the appropriate section before selecting a response.

Materials & experimental systems

n/a	Involved in the study	
x		Antibodies
x		Eukaryotic cell lines
x		Palaeontology and archaeology
x		Animals and other organisms
x		Clinical data
x		Dual use research of concern
x		Plants

Methods

n/a	Involved in the study	
x		ChIP-seq
x		Flow cytometry
x		MRI-based neuroimaging

Plants

Seed stocks	No seed stocks were produced in this study.
Novel plant genotypes	No novel plant genotypes were produced in this study.
Authentication	No seed stocks were used and no novel plant genotypes were generated in this study.
